# Simultaneous Optimization of the Ultrasonic Extraction Method and Determination of the Antioxidant Activities of Hydroxysafflor Yellow A and Anhydrosafflor Yellow B from Safflower Using a Response Surface Methodology

**DOI:** 10.3390/molecules25051226

**Published:** 2020-03-09

**Authors:** Yangyang Zhang, Li Yu, Weifeng Jin, Chang Li, Yu Wang, Haitong Wan, Jiehong Yang

**Affiliations:** 1 College of Life Science, Zhejiang Chinese Medical University, Hangzhou 310053, Zhejiang, China; zyy950909@126.com (Y.Z.); yuli9119@126.com (L.Y.); lichang@zju.edu.cn (C.L.); wangyu@zcmu.edu.cn (Y.W.); 2 College of Pharmaceutical Science, Zhejiang Chinese Medical University, Hangzhou 310053, Zhejiang, China; jin_weifeng@126.com; 3 College of Basic Medical Sciences, Zhejiang Chinese Medical University, Hangzhou 310053, Zhejiang, China

**Keywords:** hydroxysafflor yellow A, anhydrosafflor yellow B, safflower, antioxidant activity, optimization, response surface methodology

## Abstract

An evaluation of the ultrasonic extraction process and the antioxidant activities of hydroxysafflor yellow A (HSYA) and anhydrosafflor yellow B (AHSYB) from safflower are presented herein. Using response surface methodology (RSM), based on a four-factor-three-level Box–Behnken design (BBD), the extraction parameters, namely, temperature, extraction time, solvent-to-material ratio, and extraction power, were optimized for maximizing the yields of HSYA and AHSYB. The maximum yield was obtained at a temperature of 66 °C with an extraction time of 36 min, solvent-to-material ratio of 16 mL/g, and the extraction power of 150 W, which was adjusted according to the actual conditions. The HSYA and AHSYB contents were determined using high performance liquid chromatography (HPLC). The yield and the comprehensive evaluation value of HSYA and AHSYB were calculated. The antioxidant activities of the extracts were determined using a ferric reducing antioxidant power (FRAP) kit and 1,1-diphenyl-2-picrylhydrazyl (DPPH) radical scavenging activity. The results suggested that the safflower extracts possessed obvious ferric reducing and DPPH radical scavenging activities. The antioxidant activity increased with increasing concentration. The results suggested that optimizing the conditions of ultrasonic extraction using RSM can significantly increase the yields of HSYA and AHSYB from safflower. The safflower extracts showed better antioxidant activity. This study can encourage future research on cardiovascular and cerebrovascular diseases.

## 1. Introduction

**Figure 1:**
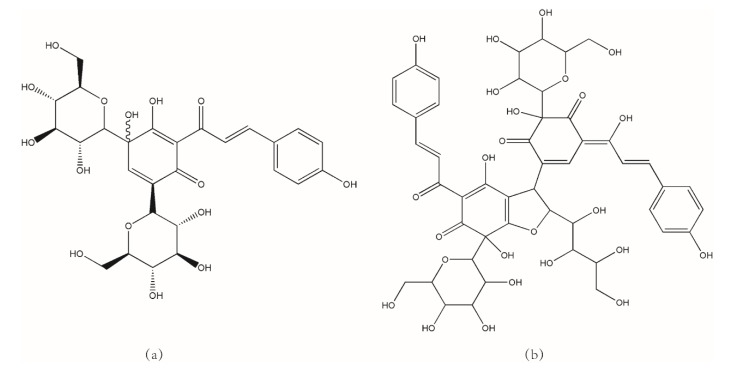
The structures of hydroxysafflor yellow A (HSYA) and anhydrosafflor yellow B (AHSYB): (**a**) HSYA and (**b**) AHSYB.

Safflower (*Carthamus tinctorius* L., Honghua in Chinese) is an annual plant belonging to the *Compositae* or *Asteraceae* family [1,2,3], which is used in Chinese folk medicine, owing to its potent bioactive properties, for the treatment of dysmenorrhea, menopause, chest pain, and abdominal pain [4,5]. It promotes blood circulation, which removes blood stasis and dredges the meridian, and can therefore be used in clinics for treating various cardiovascular and cerebrovascular diseases, including coronary heart disease, cerebral thrombosis, and myocardial ischemia [6,7]. The dry florets of the plant are the main medicinal parts. Hydroxysafflor yellow A (HSYA) (Figure 1a) is one of the most effective water-soluble bioactive ingredients of safflower, which can inhibit platelet aggregation and thrombosis, and thereby improve myocardial ischemia; it also possesses anti-oxidative and anti-inflammatory properties [8,9]. It is usually applied in food coloring additives and in the manufacture of health supplements as a safe and natural added component [10]. In particular, it has been shown to be effective in the treatment of cancer, cardiovascular diseases, cerebrovascular diseases, Alzheimer’s disease, and ischemia/reperfusion-induced acute kidney injury [11,12,13,14]. Therefore, HSYA is extensively used in the field of pharmacology. Anhydrosafflor yellow B (AHSYB) (Figure 1b) is another important bioactive ingredient [15,16,17] that is isolated from safflower. AHSYB contains numerous hydroxyl groups and possesses strong antioxidative properties; however, there are few studies on AHSYB at present. 

The extraction processes employed in traditional Chinese medicine include the decoction method, dipping method, percolation method, heating reflux method, and the steam distillation method, among others [18]. Ultrasonic extraction is a method that can accelerate the release, diffusion, and dissolution of effective substances in cells using ultrasonic cavitation, as well as mechanical and thermal effects [19]. However, the extraction yield of the compounds is influenced by various factors [20]. It has been demonstrated that temperature, extraction time, solvent-to-material ratio, and extraction power play important roles in the extraction process [21,22]. Moreover, combinations of these different factors have a significant impact on the extraction yield. The relationships among these several independent variables and the response variables are therefore explored using response surface methodology (RSM) [23]. 

RSM is a comprehensive mathematical and statistical method that has been successfully applied to multi-faceted optimization processes [24,25,26,27]. The advantage of this technology lies in the fact that fewer experiments are necessary for evaluating the effects of multiple factors and their effects on the evaluation indicators. Prior to applying the RSM, it is necessary to select an experimental design that elucidates which experiments should be performed in the study. A Box–Behnken design (BBD) is a traditional method that is used in the experimental design [28,29]. 

To date, few studies have attempted to optimize the extraction of HSYA and AHSYB from safflower using RSM. Therefore, this study aimed to build the optimized parameters for the ultrasonic extraction of HSYA and AHSYB from safflower based on the single-factor experiments and RSM. At the same time, the antioxidant activities of the safflower extracts were evaluated by determining their ferric reducing antioxidant power (FRAP) and 1,1-diphenyl-2-picrylhydrazyl (DPPH) radical scavenging activity. The results of this study may provide a reference for optimizing the extraction of HSYA and AHSYB from safflower and elucidate their potential antioxidant properties. 

## 2. Results

### 2.1. Single-Factor Experiments

Single-factor experiments were performed to determine whether the variables could be optimized for maximizing the extraction yield of HSYA and AHSYB from safflower. 

The effects of temperature (40, 50, 60, 70, and 80 °C), extraction time (20, 30, 40, 50, and 60 min), solvent-to-material ratio (10, 12, 14, 16, and 18 mL/g), and the extraction power (40, 80, 120, 160, and 200 W) were investigated for the effect on the extraction of HSYA and AHSYB. The results demonstrated that the yield of HSYA and AHSYB were increased between temperatures of 20 to 70 °C, and the values of the extraction yield reached a maximum at a temperature of 70 °C. Therefore, the temperatures of 60, 70, and 80 °C were selected for the RSM experiment. 

The same method was employed for selecting the appropriate ranges for the other variables. Three levels were selected for the aforementioned variables using single-factor experiments, with the temperature being 60, 70, and 80 °C; the extraction time being 20, 30, and 40 min; the solvent-to-material ratio being 14, 16, and 18 mL/g; and the extraction power being 120, 160, and 200 W. 

### 2.2. HPLC Analysis of HSYA and AHSYB

**Figure 2:**
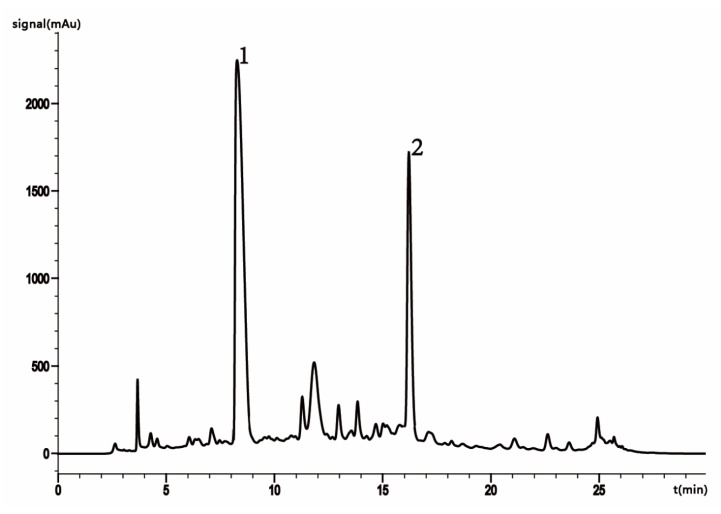
HPLC chromatogram of the extraction samples. 1: HSYA; 2: AHSYB.

The HSYA and AHSYB extracts from safflower were analyzed using HPLC and detected at a wavelength of 403 nm [30] (Figure 2). The contents of HSYA and AHSYB were determined by converting their corresponding peak areas. The peaking sequence was: HSYA > AHSYB, which means the HSYA had a lower retention time than AHSYB. The peaking sequence represents the polarity of the compound. The results show that the polarity of HSYA was greater than that of AHSYB. The content of HSYA was higher than that of AHSYB in the safflower extract. 

#### 2.2.1. Linearity

HPLC analysis was performed, as described in Section 4.4.1, using 120 µL of the control solution, as mentioned in Section 4.4.2. The regression equations were obtained by plotting the different concentrations of HSYA and AHSYB (*X*) versus the peak area (*Y*). The contents of HSYA and AHSYB were obtained by putting the peak area into the regression equation. The regression equation for HSYA was *Y* = 2.00 × 10^7^*X* − 10^6^, *R*^2^ = 0.9996, suggesting a good linearity in the concentration range of 0.30–0.70 mg/mL. The regression equation for AHSYB was *Y* = 3.00 × 10^7^*X* − 4.76 × 10^5^, *R*^2^ = 0.9997, suggesting a good linearity in the concentration range 0.08–0.16 mg/mL. 

#### 2.2.2. Precision

The precision was assessed using control solutions of HSYA (0.5 mg/mL) and AHSYB (0.1 mg/mL), where HPLC analysis was performed six times in parallel, according to the methodology described in Section 4.4.1. The precision relative standard deviation (RSD) of the peak area of HSYA was 0.91%, while that of AHSYB was 0.73%. The results demonstrated that the RSD values were less than 1% (*n* = 6), which satisfied the requirements for sample determination.

#### 2.2.3. Repeatability

Six replicas of the test solutions were prepared, as described in Section 4.3, and HPLC analysis was performed according to the method described in Section 4.4.1. The RSD of the peak areas of HSYA and AHSYB were 1.8% and 1.1%, respectively. The results indicated that the method was reproducible.

### 2.3. Effect of Variables and Model Fitting

The BBD in the optimization experiment consisted of four factors, three levels, and five central points to run, repeated three times. The three levels for each of the independent variables were denoted using −1, 0, and 1. The dependent variable was selected by considering the comprehensive evaluation values of the extraction yields of HSYA and AHSYB as the evaluation index. 

As depicted in Table 1, the results of ANOVA indicated that the model was highly significant (*p <* 0.01), but the lack of fit of each of the models was not significant. The quality of fit of the model was assessed using the determination coefficient (*R*^2^) and the adjusted determination coefficient (*R*^2^_adj_). The values of *R*^2^ and *R*^2^_adj_ were 0.857 and 0.723, respectively, and the coefficient of variation (CV) was 2.65%. The results also demonstrated that the solvent-to-material ratio (C) was highly significant (*p <* 0.01). In brief, the solvent-to-material ratio had a positive effect on the other responses. The interactive effects of the variables with the extraction time (B) and the extraction power (D) were significant (*p <* 0.05). The quadratic effect of A^2^ was significant (*p <* 0.05) and the quadratic effects of C^2^ and D^2^ were highly significant (*p <* 0.01). It could be deduced from the aforementioned factors that the response fitted the model sufficiently. The model for the comprehensive evaluation value (*Y*) is represented by the following equation:

*Y* = −6.362 + 0.038A + 0.036B + 0.612C + 9.867 × 10^−3^D − 8.694 × 10^−5^AB + 1.903 × 10^−4^AC + 8.673 × 10^−6^AD − 3.750 × 10^−4^BC − 7.331 × 10^−5^BD − 4.758 × 10^−5^CD − 2.969 × 10^−4^A^2^ − 1.917 × 10^−4^B^2^ − 0.019C^2^ − 2.344 × 10^−5^D^2^. (1)

**Figure 3:**
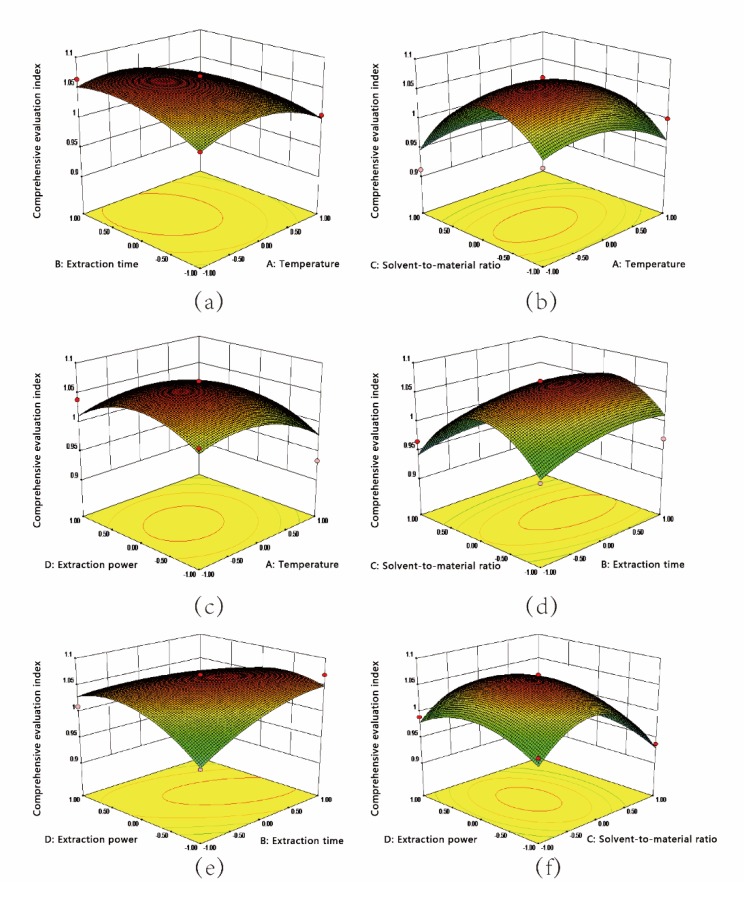
Response surfaces of the comprehensive evaluation value of HSYA and AHSYB yield: (**a**) Temperature (A) and Extraction time (B); (**b**) Temperature (A) and Solvent-to-material ratio (C); (**c**) Temperature (A) and Extraction power (D); (**d**) Extraction time (B) and Solvent-to-material ratio (C); (**e**) Extraction time (B) and Extraction power (D); and (**f**) Solvent-to-material ratio (C) and Extraction power (D).

As shown in Figure 3, the response surface 3D graphs generated using the Design Expert software were used for investigating the interaction of variables that affected the comprehensive evaluation value of the yield of HSYA and AHSYB.

### 2.4. Optimal Processing Conditions and Verification of the Predictive Model

An extraction temperature of 66.28 °C, extraction time of 35.62 min, solvent-to-material ratio of 15.62 mL/g, extraction power of 151.15 W, and a comprehensive evaluation value of 1.07 represented the optimal conditions for maximizing the extraction yields of HSYA and AHSYB. The experimentation was facilitated by slightly modifying the parameters in the verification experiments as follows: extraction temperature of 66 °C, extraction time of 36 min, solvent-to-material ratio of 16 mL/g, and extraction power of 150 W. All the experiments conducted under the optimized conditions were performed in quadruplicate and the results are depicted in Table 2. The results demonstrated that the actual values were close to the predicted values, which indicated that the model thus generated was the best model.

### 2.5. Comparison with Traditional Methods

Using the experimental conditions reported by Nie et al. [31], extraction yields of 1.28% and 0.30% were obtained for HSYA and AHYSB, respectively, and the comprehensive evaluation value was 0.78. Under the experimental conditions reported by Ji et al. [32], the extraction yields of HSYA and AHSYB were 1.64% and 0.28%, respectively. The comprehensive evaluation value was 0.93. However, the predicted comprehensive evaluation value was 1.07, and the actual comprehensive evaluation value was 1.08 under the optimal extraction conditions determined in this study. 

Comparison of the results of the above two traditional methods with the experimental results of our model revealed that the extraction yields of HSYA and AHSYB obtained under the experimental conditions used by Nie et al. and Ji et al. were lower than the extraction yields obtained under the optimized extraction conditions identified in this study. The experimental results showed that the extraction yields of HSYA and AHSYB were significantly be increased under the optimized conditions for the ultrasonic extraction process reported herein. Obviously, using our method to obtain higher yield has some practical significance for optimizing the ultrasonic extraction conditions of safflower.

### 2.6. In Vitro Antioxidant Activity

In this study, the antioxidant activities of the compounds were evaluated using the FRAP kit and DPPH radical scavenging activity [33]. The sample was prepared under the optimal extraction conditions for determining the antioxidant activity of the compounds. The antioxidant activities of the compounds are depicted in Table 3.

#### 2.6.1. FRAP

Optical density (OD) indicates the optical density absorbed by the detected object. The OD value of the FeSO_4_ standard was plotted on the abscissa, and the concentration corresponding to each OD value was plotted on the ordinate axis, in accordance with the FRAP kit instructions. The regression equation was as follows: *Y* = 0.2836*X* + 0.0118, *R*^2^ = 0.9994. The total antioxidant capacity is expressed in terms of the concentration of the standard FeSO_4_ solution. The results demonstrated that the concentration and antioxidative ferric reduction power of the sample were higher than those of the standard solution. 

#### 2.6.2. DPPH Radical Scavenging Activity

The DPPH radical scavenging activity is expressed as the rate of DPPH free radical scavenging according to the equation provided in Section 4.6.2. Analysis of the DPPH radical scavenging activity of the safflower extracts revealed that they had a notable DPPH radical-scavenging activity. The antioxidant activity increased as the concentration of the samples was increased. 

## 3. Discussion

Ethanol at different concentrations is generally used as the extraction solvent in traditional Chinese medicine. However, because as ethanol is an organic substance, also known as industrial alcohol, it is flammable, volatile, and potentially toxic. The two active ingredients (HSYA and AHSYB) extracted herein, namely HSYA and AHSYB, are water-soluble components of safflower [16]. Therefore, pure water was selected as the extraction solvent in terms of economy and safety.

During the optimization of HPLC chromatographic conditions, acetonitrile was chosen as the mobile phase because of its relatively low absorbency, low column pressure, and strong elution compared with methanol. Formic acid is more acidic and polar than acetic acid. Therefore, in this experiment, acetonitrile and 0.1% formic acid in water were used as the mobile phase, which improved the resolution of HSYA and AHSYB (R > 1.5). As the UV absorption of HSYA and AHSYB is high at a wavelength of 403 nm, HPLC analysis was performed at a wavelength of 403 nm.

Pharmacological studies have demonstrated that safflower extracts have a plethora of activities, including antiplatelet, anti-ischemic, antioxidative, and anti-inflammatory properties [34]. HSYA and AHSYB can possibly induce a combined antioxidative activity by scavenging the free radicals and inhibiting lipid peroxidation [35]. Therefore, the extraction of HSYA and AHSYB from safflower using the ultrasonic extraction strategy could be important for the antioxidant activity.

The results of ANOVA demonstrated that the extraction time and extraction power (BD) had a significant (*p* < 0.05) negative effect on the comprehensive evaluation value (Table 1). The coefficient of variation (CV) is defined as the quotient of standard deviation and mean value [36,37]. CV was used for analyzing the statistical data. The value of CV is related to the reproducibility of experiments. A lower value of CV indicates a smaller variation in the mean value and the better reproducibility of the experiment [38]. Values of CV < 10% indicate the reliability and accuracy of the experiment [39,40]. In our study, the value of CV was 2.65%, which indicates that the RSM was more consistent with the experimental results.

The comprehensive evaluation value gradually increased as the extraction power was increased over a shorter extraction time. However, the comprehensive evaluation value decreased as the extraction power was increased over a longer extraction time (Figure 3e). This effect allowed for the multi-level effects of load backgrounds and mechanical vibrations induced by ultrasonic radiation pressure, which can accelerate solvent penetration and improve the extraction efficiency [41]. In our study, the extraction yields of HSYA and AHSYB increased with increasing temperature, but decreased when the temperature was increased beyond a certain value. High temperatures cause a decrease in the cavitation effect owing to an increase in the vapor pressure and a reduction in the viscosity and surface tension of the solvent, which reduces the extraction efficiency [42,43].

In our study, the extraction yields of HSYA and AHSYB increased with an increase in the solvent-to-material ratio. This was because the dissolution of the compounds increased as the solvent-to-material ratio was increased. Generally, higher solvent-to-material ratios result in greater differences in the concentration, which facilitates the diffusion of components into the solvent and accelerates mass transfer. However, after the solvent-to-material ratio has been increased to a certain extent, both HSYA and AHSYB dissolve in the solvent and the solution is near to saturation, following which, the increase in the amount of dissolution is slow. Therefore, as the solvent-to-material ratio is increased, the extraction yields of HSYA and AHSYB increase at first and then decrease.

Determination of the antioxidant activity revealed that the safflower extracts could serve as effective antioxidant agents. In our study, the antioxidant activity of safflower extract was determined using FRAP and DPPH at concentrations of 2.19 mg/mL, 4.38 mg/mL, 8.75 mg/mL, 17.50 mg/mL, and 35.00 mg/mL. At the concentration of 2.19 mg/mL, the absorbance of safflower extract was the same as that of the standard solution of 0.35 mM FeSO_4_, and the total antioxidation ability was 0.35 (mM/(1 mg/mL)), and DPPH radical scavenging percentage was 11.63%. When the concentration of safflower extract was increased to 35 mg/mL, the total antioxidation ability increased to 3.01 (mM/(1 mg/mL)), and the DPPH radical scavenging percentage was 41.90%. The results showed that the safflower extract had a weak antioxidant activity at low concentrations and the antioxidant activity increased linearly as the increase of concentration. The results of the antioxidant assay were consistent with the experimental results reported by Yao et al. [6]. The antioxidant activity increased with the increase of the concentration of safflower extract; meanwhile, the contents of HSYA and AHSYB in high concentration safflower extracts were also relatively high [16,44]. This indicates that HSYA and AHSYB played an important role in the antioxidant activity of the safflower extract. 

The results of this study demonstrated that optimizing the conditions of ultrasonic extraction using RSM can significantly increase the extraction yields of HSYA and AHSYB from safflower. In the future, we intend to study the pharmacological effects and mechanisms of AHSYB, both in vivo and in vitro, in cerebral ischemic diseases.

## 4. Materials and Methods

### 4.1. Materials

The dry florets of *C. tinctorius* L. (the dry matter content was 88%) were purchased from Hangzhou Huiyuan Industrial Co., Ltd. (Hangzhou, China) of Zhejiang Chinese Medical University and identified by Shenwu Huang, Professor at Zhejiang Chinese Medical University. The HSYA reference substance was purchased from Nanjing Shizhou Biotechnology Co., Ltd. (Nanjing, China) and the AHSYB reference substance was purchased from Shanghai Yuanye Biotechnology Co., Ltd. (Shanghai, China). HPLC grade methanol and acetonitrile were purchased from Tedia Company, Inc. (Fairfield, OH, USA). Deionized water was prepared using a Millipore water purification system (Millipore Co., Ltd., Billerica, MA, USA), and an ultrasonic cleaner (KQ5200DE, Kunshan Ultrasonic Instrument Co., Jiangsu, China). The FRAP kit was purchased from Nanjing Institute of Bioengineering (Nanjing, China), and DPPH was purchased from Shanghai Biochemical Co., Ltd. (Shanghai, China). All the other solvents used in the experiments were of analytical grade.

### 4.2. Response Surface Methodology

#### 4.2.1. Variables Selection

The extraction yield of traditional Chinese medicines is affected by numerous factors, including the extraction temperature, time, solvent concentration, solvent-to-material ratio, pH value, ultrasonic power, particle size, and the ultrasonic extraction [45]. The temperature, extraction time, solvent-to-material ratio, and extraction power were selected as variables based on the single-factor experiments. Particle size is a factor that is known to affect the yield of compounds during extraction [21,46]. Therefore, a particle size of 100 mesh was used in our study.

In our study, the three levels of variables were determined using the extraction yields of HSYA and AHSYB. First, the safflower was extracted under five levels of variables, and the level with the highest extraction yield was selected. Then, each level either side of the highest level was selected in order to determine the three levels of the variable. The three levels of the four factors were all determined using the same method.

#### 4.2.2. BBD for Extraction Optimization

In the present study, a four-factor-three-level BBD was employed for optimizing the extraction conditions using RSM [47]. The four independent variables were temperature (°C, A), extraction time (min, B), solvent-to-material ratio (mL/g, C), and extraction power (W, D). The three levels for each of the independent variables were denoted as −1, 0, and 1, The coded and actual levels of the independent variables are listed in Table 4. The dependent variable was selected by considering the comprehensive evaluation values of the extraction yields of HSYA and AHSYB as the evaluation index in accordance with the central combination experimental design principle of BBD. The number of experimental groups selected in this study was 30 groups with each group comprising 5 g of safflower (*n* = 3). The scheme of analysis and the results of the experiments are provided in Table 5. The optimization conditions were predicted using the following second-order polynomial regression model:

$$Y=\beta_{0}+\overset{4}{\underset{i=1}{\sum }}\left(\beta_{i}x_{i}\right)+\overset{3}{\underset{i=1}{\sum }}\overset{4}{\underset{j=i+1}{\sum }}\left(\beta_{ij}x_{i}x_{j}\right)+\overset{4}{\underset{i=1}{\sum }}\left(\beta_{ii}x_{i}{}^{2}\right).$$

### 4.3. The Ultrasonic Extraction Process

Ultrasonic extraction was performed in an ultrasonic cleaner equipped with a timing device and a digital temperature controller, and fitted with a temperature display. Five grams of the dry powder of safflower was placed into a 250 mL conical flask containing water at the different solvent-to-material ratios, and was subsequently placed in a conical flask in an ultrasonic cleaner. The extraction conditions in terms of the temperature, extraction time, and extraction power were selected by performing single-factor experiments. The extracts were filtered using a Buchner funnel, and the filtrate was centrifuged at 24,148.8× *g* for 10 min. The supernatant was subsequently passed through a 0.22 µm microporous membrane. Finally, the filtrates were subjected to HPLC. The ultrasonic extraction method employed herein for the extraction of HSYA and HSYB from safflower was straightforward, and the extraction time was reduced in our study.

### 4.4. HPLC Analysis

#### 4.4.1. Chromatographic Conditions

The contents of HSYA and AHSYB were determined using an Extend-C_18_ Column (4.6 mm × 250 mm, 5 µm) with a Waters 2695 HPLC system equipped with an online vacuum degasser, autosampler system (Waters, Puerto Rico, USA), column oven, 2498 UV detector (Waters, Puerto Rico, USA), and the Empower software (Waters, Puerto Rico, USA). The mobile phase consisted of acetonitrile (A) and 0.1% formic acid in water (B), and the following gradient elution strategy was used: 0–12 min, 10%–22% A; 12–20 min, 22%–26% A; and 20–30 min, 26%–95% A. The flow rate was 1 mL/min and the temperature was 25 °C. The separation was detected using UV at a wavelength of 403 nm. The injection volume was 10 µL.

#### 4.4.2. Preparation of Control Solutions

The reference substances of HSYA and AHSYB were weighed accurately and dissolved in deionized water for preparing the individual store solutions with a concentration of 1 mg/mL. A series of standard working solutions of HSYA with concentrations of 0.30, 0.40, 0.50, 0.60, and 0.70 mg/mL were prepared from the store solution by adding deionized water. The reference solutions for AHSYB with concentrations of 0.08, 0.10, 0.12, 0.14, and 0.16 mg/mL were prepared using the same method that was employed for preparing the reference solutions for HSYA. 

### 4.5. Calculation of the Comprehensive Evaluation Value

The weight coefficients of HSYA and AHSYB were 0.478 and 0.522, respectively, as determined using the entropy weight method [48,49]. The following equation was used for calculating the comprehensive evaluation value (*Y*): *Y* = 0.478*Y*_1_ + 0.522*Y*_2_. The comprehensive evaluation values are listed in Table 2.

### 4.6. Determination of Antioxidant Activity

#### 4.6.1. FRAP Assay

The total antioxidant capacity was evaluated using the FRAP kit [50]. A fresh working solution was prepared by mixing 3.750 mL of a detection buffer, 0.375 mL of matrix fluid, and 0.375mL of a substrate solution, and incubated for 10 min at 37 °C in the dark. A fresh solution of 100 mM FeSO_4_ was prepared using FeSO_4_·7H_2_O as the standard material. The test solution was prepared by adding 180 µL of FRAP reagent and 5 µL of the sample solution. The blank control was prepared using 180 µL of FRAP reagent and 5 µL of distilled water. The absorbance was measured at 593 nm. The total antioxidant capacity was expressed as the concentration of the standard solution of FeSO_4_. 

#### 4.6.2. Measurement of DPPH Radical Scavenging Activity 

The DPPH radical scavenging activity was measured as previously described [51] with some modifications. The solution of DPPH radicals was prepared by adding 180 µL of 0.15 mM DPPH in anhydrous ethanol and 2 µL of the sample solution at different concentrations. The mixture was subsequently incubated for 30 min at room temperature in the dark, and the absorbance of the solution was measured at 517 nm. The DPPH radical scavenging activity was calculated according to the following equation:

$$\mathrm{scavenging}\;\mathrm{percentage}\;\left(\%\right)=\left(1-\frac{A_{2}-A_{0}}{A_{1}}\right)\times 100\%,$$

where *A*_0_ is the absorbance of sample + anhydrous ethanol, *A*_1_ is the absorbance of anhydrous ethanol + DPPH, and *A*_2_ is the absorbance of sample + DPPH.

### 4.7. Statistical Analyses

All the measurements were obtained from experiments performed in triplicate. The BBD was determined using Design Expert software, version 8.0.6 (Stat-Ease Inc., Minneapolis, MN, USA). The *p*-values less than 0.05 or 0.01 were considered to be statistically significant.

## 5. Conclusions

In this study, RSM was employed for optimizing the extraction parameters of two active components, namely HSYA and AHSYB, from safflower based on the single-factor experiments. The optimal extraction conditions, adjusted according to the actual conditions, were: temperature of 66 °C, extraction time of 36 min, solvent-to-material ratio of 16, and ultrasonic power of 150 W. The results of the model validation demonstrated that the predicted values agreed with the predicted values, suggesting that the second-order model developed for increasing the extraction yields of HSYA and AHSYB from safflower was accurate and reliable. The results of the antioxidant activity assay suggested that the safflower extracts possessed antioxidant activity, and the antioxidant capacity of the safflower extracts increased with increasing concentration. This study aimed to optimize the extraction method for safflower, and we intend to investigate the antioxidant properties of safflower in future studies. Such studies are necessary as it is essential to obtain more information pertaining to traditional Chinese medicine.

## Figures and Tables

**Table 1:** Analysis of variance and significance test.

Source	Sum of Squares	Degree of Freedom	Mean Square	f-Value
Model ^a^	0.063	14	4.508 × 10^−3^	6.400 ^**^
A	2.946 × 10^−3^	1	2.946 × 10^−3^	4.180
B	1.297 × 10^−3^	1	1.297 × 10^−3^	1.840
C	7.123 × 10^−3^	1	7.123 × 10^−3^	10.110 ^**^
D	2.528 × 10^−6^	1	2.528 × 10^−6^	3.587 × 10^−3^
AB	3.023 × 10^−4^	1	3.023 × 10^−4^	0.430
AC	5.795 × 10^−5^	1	5.795 × 10^−5^	0.082
AD	4.814 × 10^−5^	1	4.814 × 10^−5^	0.068
BC	2.250 × 10^−4^	1	2.250 × 10^−4^	0.320
BD	3.440 × 10^−3^	1	3.440 × 10^−3^	4.880 ^*^
AD	5.795 × 10^−5^	1	5.795 × 10^−5^	0.082
A^2^	6.046 × 10^−3^	1	6.046 × 10^−3^	8.580 ^*^
B^2^	2.521 × 10^−3^	1	2.521 × 10^−3^	3.580
C^2^	0.041	1	0.041	58.120 ^**^
D^2^	9.648 × 10^−3^	1	9.648 × 10^−3^	13.690 ^**^
Residual	0.011	15	7.047 × 10^−4^	
Lack of fit	0.011	10	1.054 × 10^−3^	157.100
*R* ^2^				0.857
*R* ^2^_adj._				0.723
Std. Dev.				0.027
Mean				1.00
CV (%)				2.65

^a^ A: temperature (°C); B: extraction time (min); C: solvent-to-material ratio (mL/g); D: extraction power (W). CV: Coefficient of variation. Level of significance: * *p* < 0.05, indicates significant; ** *p* < 0.01, indicates highly significant.

**Table 2:** Verification test under optimized conditions from the response surface.

Verification Experiment	Extraction Yield (%)		Comprehensive Evaluation Value (*Y*)	Average Value
	HSYA (*Y*_1_)	AHSYB (*Y*_2_)		
1	1.80	0.42	1.08	1.08
2	1.81	0.40	1.07	
3	1.82	0.42	1.09	
4	1.82	0.42	1.09	

**Table 3:** The results of antioxidant activities. DPPH: 1,1-diphenyl-2-picrylhydrazyl, FRAP: Ferric reducing antioxidant power.

	Concentration of Safflower Extract (mg/mL)				
Antioxidant Activity	2.19	4.38	8.75	17.50	35.00
FRAP (mM/(1 mg/mL))	0.35	0.65	0.99	1.79	3.01
DPPH Radical Scavenging Activity (%)	11.63%	15.71%	20.52%	28.49%	41.90%

**Table 4:** Independent variables and their levels used in the Box–Behnken design (BBD).

Factors	Levels		
	−1	0	1
Temperature (°C, A)	60	70	80
Extraction Time (min, B)	20	30	40
Solvent-to-Material Ratio (mL/g, C)	14	16	18
Extraction Power (W, D)	120	160	200

**Table 5:** Box–Behnken design (BBD) for the independent variables and corresponding response values.

Run	Temperature (°C, A)	Extraction Time (min, B)	Solvent-to-Material Ratio (mL/g, C)	Extraction Power (W, D)	Extraction Yield (%)		Comprehensive Evaluation Value (*Y*)
					HSYA (*Y*_1_)	AHSYB (*Y*_2_)	
1	1	0	0	−1	1.55	0.37	0.93
2	0	0	0	0	1.78	0.42	1.07
3	0	−1	1	0	1.65	0.34	0.97
4	0	0	1	1	1.59	0.32	0.93
5	0	0	0	0	1.77	0.42	1.07
6	0	0	1	−1	1.58	0.35	0.94
7	1	1	0	0	1.72	0.38	1.02
8	−1	0	0	−1	1.72	0.39	1.03
9	1	0	0	1	1.63	0.35	0.96
10	1	−1	0	0	1.71	0.36	1.01
11	0	0	0	0	1.78	0.41	1.06
12	−1	1	0	0	1.78	0.41	1.06
13	1	0	1	0	1.59	0.34	0.94
14	0	−1	0	−1	1.64	0.35	0.97
15	−1	0	−1	0	1.68	0.36	0.99
16	0	0	0	0	1.77	0.42	1.07
17	0	1	0	1	1.68	0.37	1.00
18	−1	0	0	1	1.76	0.38	1.04
19	0	−1	0	1	1.71	0.37	1.01
20	0	−1	−1	0	1.67	0.33	0.97
21	1	0	−1	0	1.69	0.37	1.00
22	0	1	−1	0	1.66	0.34	0.97
23	−1	0	1	0	1.57	0.31	0.91
24	0	0	−1	−1	1.67	0.36	0.99
25	0	0	0	0	1.78	0.42	1.07
26	0	1	0	−1	1.79	0.41	1.07
27	0	1	1	0	1.61	0.32	0.94
28	0	0	−1	1	1.69	0.35	0.99
29	0	0	0	0	1.78	0.41	1.06
30	−1	−1	0	0	1.73	0.36	1.01
